# Topology-Controlled Photophysics of Ionized C_14_H_10_ Isomers

**DOI:** 10.1021/acs.jpca.6c04463

**Published:** 2026-09-21

**Authors:** Anna Maria Reider, Miriam Kappe, Paul Martini, Martin Schmidt, Serge A. Krasnokutski, Paul Scheier

**Affiliations:** † 27255 University of Innsbruck, Technikerstrasse 25, Innsbruck 6020, Austria; ‡ 7675 University of Stockholm, AlbaNova, Roslagstullsbacken 21, Stockholm 11421, Sweden; § 9378 University of Jena, Helmholtzweg 3, Jena 07743, Germany

## Abstract

The optical signatures of polycyclic aromatic hydrocarbons (PAHs) are highly sensitive to their molecular geometry, charge state, and intermolecular interactions. A comparison between the linear anthracene and angular phenanthrene ions highlights how topological effects govern the degree of electronic delocalization in the excited state, resulting in spectroscopic signatures of vibronic coupling, varying oscillator strengths, and nonradiative relaxation pathways. This work presents the UV–vis spectra of the cationic, protonated, and anionic C_14_H_10_ isomers utilizing He-tagging action spectroscopy via helium nanodroplet isolation. The linear anthracene cations retain a well-resolved vibronic structure, characteristic of delocalized π-systems, whereas the angular phenanthrene cation produces broader bands typical of enhanced vibronic coupling and a more localized charge distribution. Hydrogenation disrupts conjugation and quenches the vibronic progression most strongly in anthracene. Notably, the broad, weakly structured spectrum of protonated phenanthrene is consistent with the pronounced excited-state twisting predicted by previous calculations. Anionic C_14_H_10_, on the other hand, displays weak continua, aligning with low oscillator strengths and rapid autodetachment. Dimerization generally broadens and weakens the absorption features, particularly for phenanthrene, consistent with the coexistence of several low-lying π-stacked isomers and enhanced vibronic coupling. Comparison with matrix isolation and gas-phase spectra supports the high fidelity of the helium nanodroplet approach.

## Graphical abstract

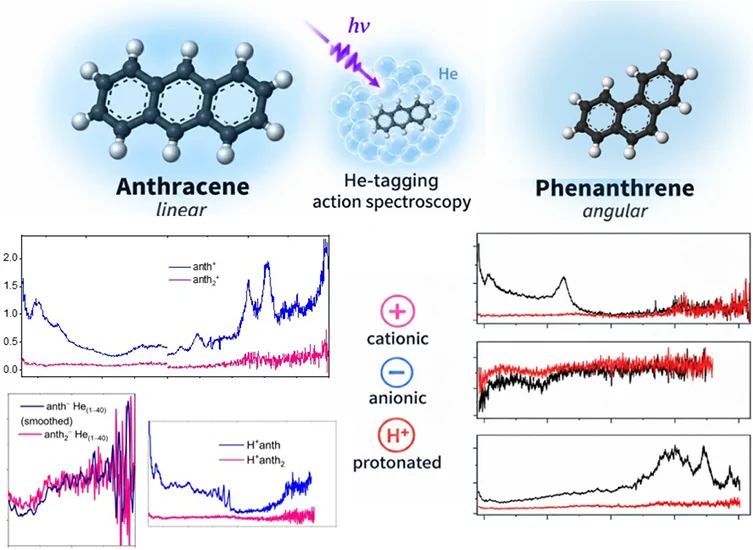

## Introduction

Polycyclic aromatic hydrocarbons (PAHs) are of particular interest across the diverse fields of chemical physics, including combustion and environmental chemistry^1–5^ molecular materials science^6–8^ and astrochemistry.^9–15^ Especially in the latter field, their neutral and ionic forms are proposed as carriers of diffuse interstellar bands (DIBs), the extended red emission (ERE), and as contributors to the infrared emission features of the interstellar medium (ISM).^5,9,11,16–19^ As purely π-conjugated systems, their chemical reactivity, stability, and optical and electronic behavior are directly governed by their exact geometry and the resulting degree of electronic delocalization within the aromatic framework.^8,20–23^ Removal or addition of a charge redistributes the π-electron density, typically leading to partial localization and enhanced vibronic coupling that drastically changes the shape, intensity and lifetime of optical transitions. The extensive electronic reorganization is further modified as ions assemble into clusters, and intermolecular π-π interactions reshape their electronic signatures. Understanding how the molecular structure shapes the spectroscopic properties of PAHs is therefore of broad relevance to both fundamental science and the interpretation of astrophysical spectra. This study focuses on two relatively small members of the extended PAH family, namely anthracene (anth) and phenanthrene (phen), which are isomers of C_14_H_10_. As prototypical models for PAHs, anth is linear with D_2_h symmetry and an extended conjugation along a single axis, whereas the kinked, angular structure of phen results in C_2_v symmetry and a more localized electronic density. The increased π-bonding interactions, i.e., the larger aromaticity, make phen more stable than anth, an effect that can drastically change upon ionization.^23,24^ Removal of an electron redistributes the π-electron density and alters the bond-length patterns, often under conservation of the molecular symmetry, while protonation additionally disrupts the π-conjugation and generally lowers the molecular symmetry,^20,25,26^ The stability of anionic species, however, is largely governed by their electron affinity (EA) and the competition between electron detachment and stabilization through solvation or complexation.^27^ While the smallest (poly-)­aromatic hydrocarbons (benzene and naphthalene) have negative EAs, larger PAHs have positive EAs, starting with anth and phen.^28–35^ The EA of phen is particularly low and, for a long time, has been argued to be even negative, which makes the detection of phen^–^ challenging.^36–40^ One reliable approach for the stabilization of phen^–^ is its complexation with a ligand, such as He.^28,41^ The lifetime of a He_*x*_phen^–^ complex with *x* as low as *x* = 1 is then sufficiently long for experimental observations. In contrast to the weakly bound anionic phen^27,33,36,42^ the more positive electron affinity of anth results in a bound radical-anion ground state with the excess electron delocalized over its extended π framework.^33,35,42–46^ The earliest research on the C_14_H_10_ PAH dates back to the 1950s and 1960s, with absorption and fluorescence spectra of neutrals and ab initio molecular orbital calculations, revealing fundamental electronic and vibrational states and the impact of dimerization and environment-induced modifications in mixed crystals, thereby providing the groundwork for later experimental and theoretical studies.^47–53^ When matrix isolation spectroscopy became a feasible experimental approach around 1990, the focus shifted to more detailed, theory-based insights into radical cations, revealing how ionization lowers symmetry, alters bond-length patterns, and shifts absorption bands.^25,54–60^ More recent works have extended these approaches to protonated or hydrogenated species and condensed-phase environments, linking PAH ions to astrochemical phenomena and revealing how their geometry influences their reactivity and stability in various environments.^20,26,29,61–65,65^

Despite these advances, systematic spectroscopic comparisons across charge states and clustering of PAHs remain rare. Existing studies have predominantly focused on neutrals or radical cations, often matrix-bound, leaving the interplay between geometry, charge localization, and π-stacking largely unexplored. Action spectroscopy in helium nanodroplets (HNDs) offers a potent diagnostic tool to address this gap. The ultracold, almost perturbation-free helium environment enables the generation of both cationic and anionic anth and phen oligomers, with the consecutive He-tagging spectroscopy providing insights into the molecular signatures of their monomers and dimers with minimal spectral broadening and matrix shift. We therefore present an extensive comparison between cationic and protonated anth and phen, and additionally report trend-level spectral signatures of their dimers and a comparison with the intrinsically weaker anionic phenanthrene signal. By contrasting the two isomeric frameworks in different charge states, this study establishes a direct link between molecular geometry and the UV–vis response of charged aromatic clusters.

## Methods

All measurements are carried out on our ClusTOF instrument.^66^ Superfluid helium nanodroplets (HNDs), each containing several million He atoms, are generated by expanding precooled (9–10 *K*), high-purity helium (He 6.0 Messer, 30 bar) through a 5 μm nozzle into ultrahigh vacuum.^67,68^ After passing through a skimmer, the HNDs are ionized by electron impact, producing multiply charged cationic (65–68 eV, 140–240 μA) or anionic (24 eV, 240–280 μA) droplets.^69–71^ Charge centers, predominantly in the form of He_3_^+^ (about +10e per HND) or He^*–^ (up to −2e per HND), reside near the droplet surface and act as nucleation sites for dopant aggregation.^71,72^ Anthracene (anth) or phenanthrene (phen) (both from Sigma-Aldrich, 99% purity) is introduced into a differentially pumped vacuum chamber via resistively heated reservoirs (below 30 °C for phen and approximately 70 °C for anth). When the HND picks up these molecules from the gas phase, ion-induced dipole interactions guide the dopants to the charge centers. Charge transfer then produces ionic C_14_H_10_ clusters. To achieve hydrogenation of these aggregates, H_2_ gas (Messer, 99.999% purity) is introduced via a gas inlet into the vacuum chamber where the droplets are ionized. In this scenario, the charge centers initially formed are H_3_^+^(H_2_)_m_, which subsequently act as proton donors to the introduced PAHs in the ultracold HND.^73^ To release the clusters from the He matrix, the HNDs collide with a stainless-steel surface,^66^ liberating the He-tagged complexes that are subsequently detected with our time-of-flight (ToF) mass spectrometer (HTOF, Tofwerk AG). A typical mass spectrum has a resolving power of m/Δm ≈ 2000 (cations), 1700 (protonated), and 1600 (anions). Action spectroscopy is performed by irradiating the He-tagged ions with a tunable, pulsed laser (EKXPLA NT242-SH: 210–2600 nm, line width <5 cm^–1^, maximal pulse energy 450 μJ). With the 1 kHz laser synchronized to every tenth extraction pulse of the 10 kHz ToF, spectra are obtained by comparing the ion yields with and without photon interaction. Resonant absorption leads to He evaporation, observed as depletion of the (C_14_H_10_)_*m*_^±^ He_*n*_ signal accompanied by an increase of the bare (C_14_H_10_)_*m*_^±^ signal. The absorbance *A* is defined via the natural logarithm of the laser-on (*I*_laser_)/laser-off (*I*_off_) ion ratio, corrected for background (*I*_BG_, summed signal over the mass spectrum when the laser is off):

1 $$A=-\ln\left(1-\frac{I_{\mathrm{laser}}-I_{\mathrm{off}}}{I_{\mathrm{BG}}}\right)$$

With the convention of the sign of this calculation, positive values correspond to absorption (increase in the parent ion) and negative intensities denote depletion (electron detachment or loss of He tags). The data are further normalized for wavelength-dependent changes in the laser power by assuming a direct correlation between laser power and the number of photons (*N*). The final ion yield (*Y*) is hence presented as

2 $$Y=10^{20}\times \frac{A}{N}$$

Calculations of geometries, energies, and vibrational frequencies of cationic and anionic dimers were performed at the cam-b3lyp+D3/6–311+g­(d,p) level of theory,^74^ employing D3 dispersion correction as suggested by Grimme.^75^ Electronic states were calculated at the time-dependent DFT level using the TD-cam-b3lyp+D3/6–311+g­(d,p) level of theory. All calculations were performed using Gaussian software.^76^

## Results and Discussion

The cationic and protonated spectra of anth were reported previously.^63,77^ Here, they are complemented by anionic spectra and used as a reference for the comparison to the spectra of phenanthrene to address how the molecular topology of the two C_14_H_10_ isomers influences the spectroscopic signatures in their cationic, protonated, and anionic forms.

### Anionic Anthracene

Absorption spectra of anionic anth were first obtained at room temperature in glassy solutions^78^ and at lower (77K) temperature glassy matrices.^79^ Early DFT studies of PAH anions predict a series of π → π* transitions in the visible and near-UV.^44^ More recent experimental and theoretical works, however, found that many of these excited states are not strongly bound, but rather correspond to resonant states embedded in the autodetachment continuum with varying lifetimes and possible internal conversions between resonances.^35,46,80^ Additional studies on anth cluster anions suggest that the excess electron in anth_2_^–^ is evenly delocalized over the two anth units and can be interpreted as a dimeric anion rather than an anion-solvent.^45^ The depletion spectrum of anth^–^He_1–40_ and anth_2_^–^He_1–40_ in Figure 1 can be interpreted as a representation of these resonance manifolds with an overall energy range that agrees with the features found by photoelectron spectroscopy and DFT calculations.^46,80^

**Figure 1:**
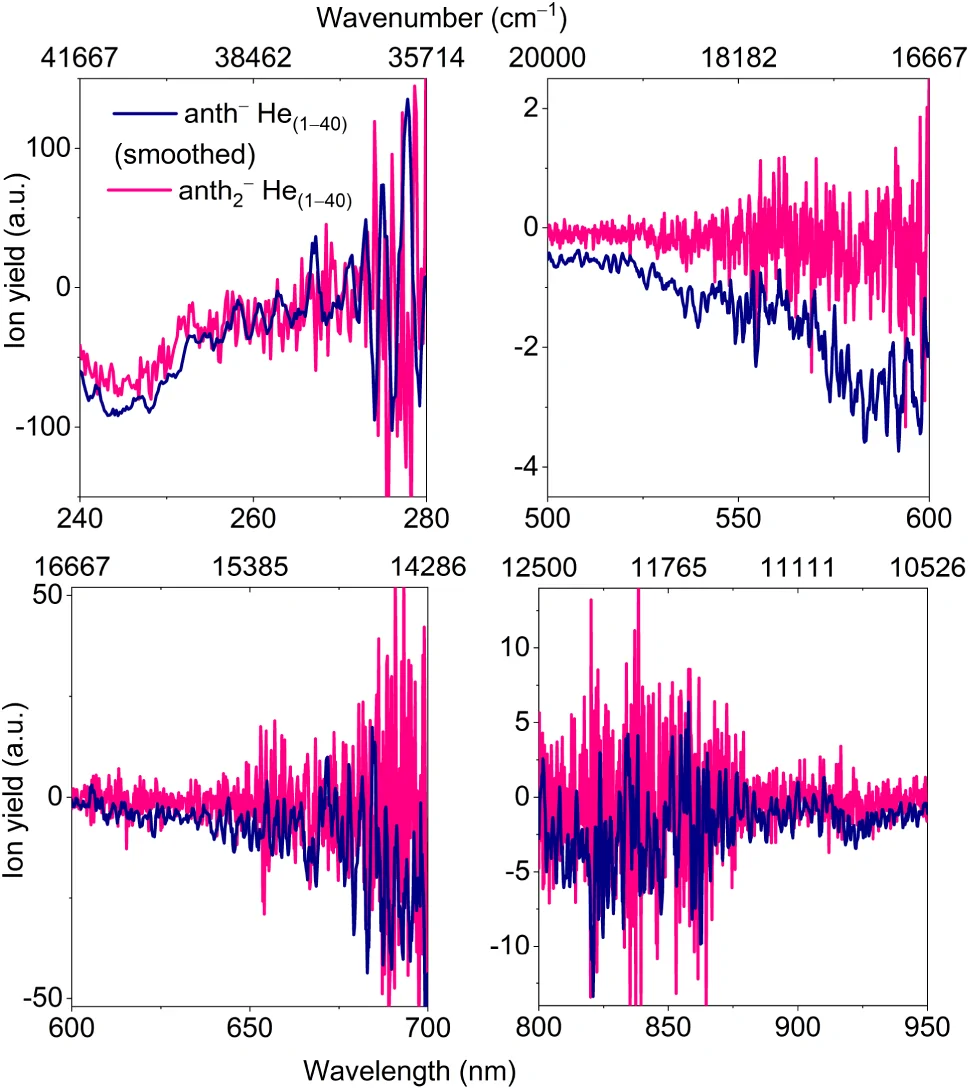
Experimental spectra of the anionic, He-tagged anth complexes (anth^–^He_(1–40)_ blue data, anth_2_^–^He_(1–40)_ magenta data).

### Ionized Phenanthrene

The He-tagging action spectroscopy allows observation of the absorption by the cationic and protonated parent species (signal on untagged phen) in the upper two panels of Figures 2–3. The signal from the anionic complex is detectable solely in the depletion of the He-tagged phen, as shown in the lower panels of the same figures. In contrast to the pure π-systems, the protonated complexes, H^+^phen, provide insight into how π-electron disruption influences the electronic signatures. The absorption spectra of the species are charge-state dependent, with cationic phen displaying intense, structured π → π* bands that partially persist upon dimerization. In contrast, the protonated ions exhibit broader, red-shifted absorption envelopes consistent with partial π-disruption and increased vibronic coupling. The anionic spectra are generally governed by low oscillator strengths and possibly rapid autodetachment, leading to weak signals and broad continua rather than discrete bands. All peaks are summarized according to charge state in Table 1 along with comparisons with values reported in the literature, where available.

**Figure 2:**
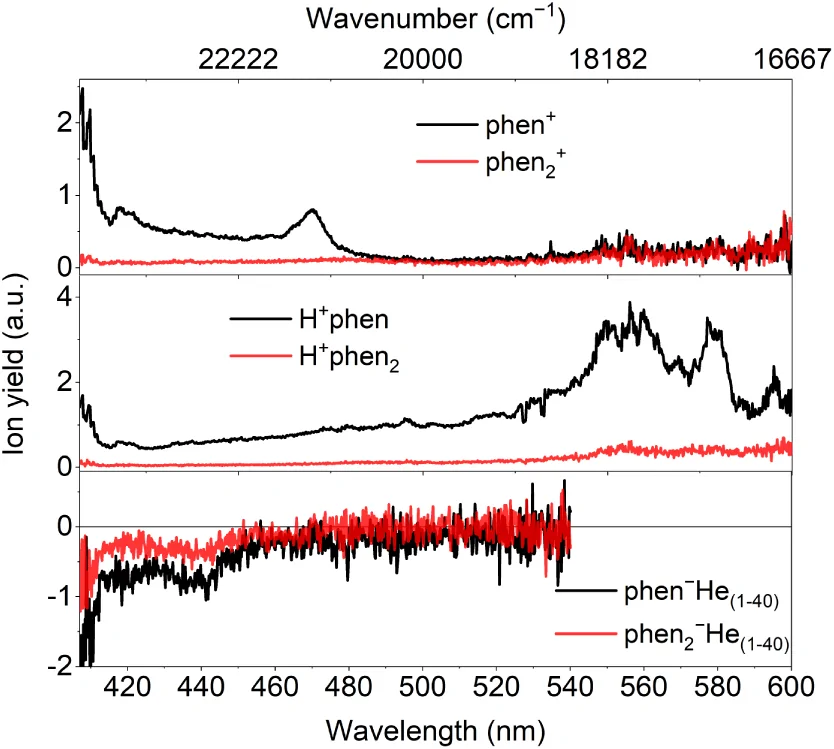
Experimental spectra in the range of 405–600 nm. The upper panel presents the cationic phen^+^ (black) and phen_2_^+^ (red), the middle panel the corresponding protonated species H^+^phen, and the lowest panel the summed He-tagging signal of the anion, phen^–^He_(1–40)_.

**Figure 3:**
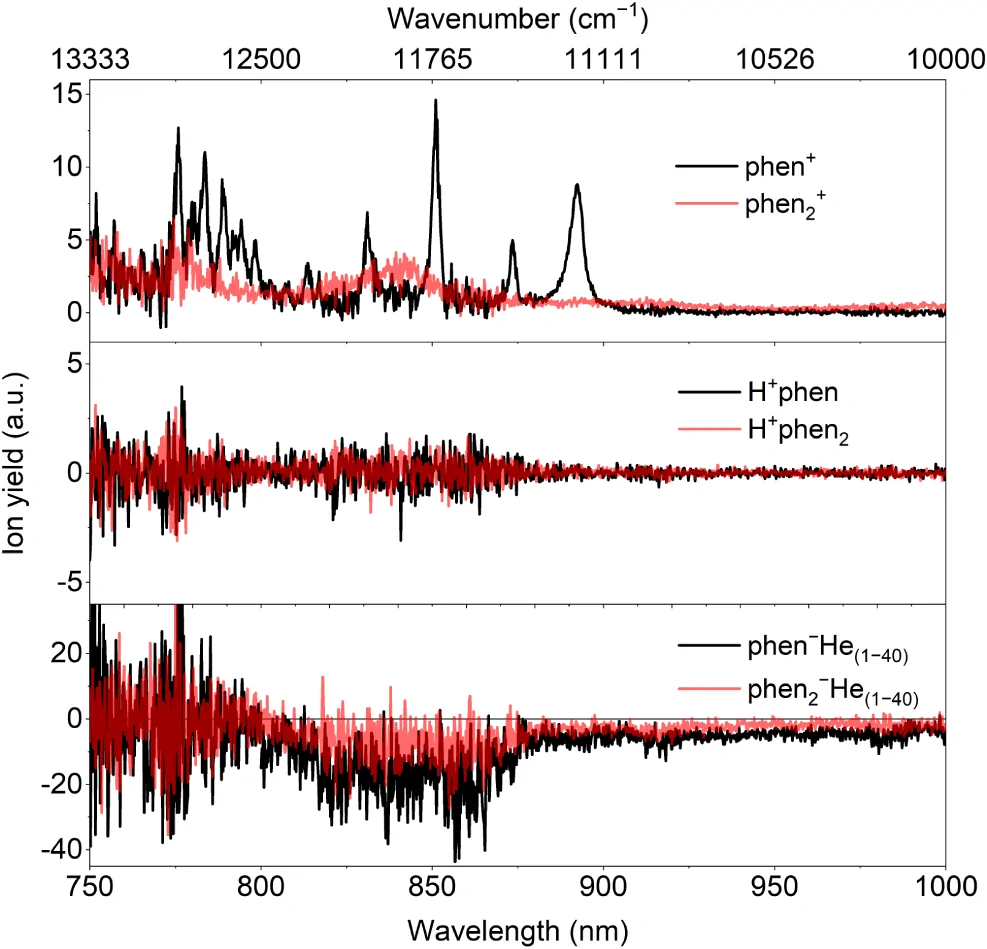
Experimental spectra in the range of 750–1000 nm. The spectra of the dimers (red data) are drawn transparently to improve visibility of the overlying monomer spectra.

**Table 1:** Experimentally Confirmed Peaks from This Work in Comparison to Available References from the Literature via Matrix Isolation spectroscopy^26,56,58^ and Gas-Phase Photofragment Spectroscopy^85^

Cationic phen		Protonated phen	
Exp. (nm)	Ref. (nm)	Exp. (nm)	Ref. (nm)^26^
892.4	899.8,^56^ 898.3,^58^ 891.9 ^85^	777	
873.4	880.6,^58^ 873.3 ^85^	646	
851.0	857.8,^56^ 856.6,^58^ 850.8 ^85^	640	
831.1	837.8,^56^ 836.8,^58^ 834.6 ^85^	615.5	
816.9	820.0,^56^ 818.9,^58^ 816.6 ^85^	595	596.6
813.5	813.4 ^85^	586	583.3
807.8		579	
802.8		577	578.2
798.3	802,^56^ 801.4,^58^ 798.1 ^85^	573	570
794.2	802,^56^ 797.6,^58^ 794.0 ^85^	569	
789.0	795,^56^ 794.5 ^58^	563	564.7
783.5	789.2,^56^ 787.4,^58^ 783.3 ^85^	560	561.4
775.7	781.6,^56^ 780.4,^58^ 775.7 ^85^	556	
756.9	762.5 ^56^	554	
752.0	756.8,^56^ 755.2 ^58^	550	
470.2	473.7,^56^ 460.3 ^58^	541	543.7
418		418	

Figure 2 presents the wavelength range from 405 to 600 nm (16667–23810 cm^–1^), where all investigated complexes share a common sharp increase in absorption toward the blue with an overlying structure on the cationic and protonated phen absorption. The absorption of anionic phen^–^He_(1–40)_ is detectable via depletion (electron or He detachment). The cation spectrum shows broader absorption peaks at 420 and 470 nm, and some additional weaker features between 535 and 600 nm, which are also preserved in the dimer signal, phen_2_^+^. The protonated complex shares the absorption at 418 nm, followed by a gradually increasing signal without any relevant features until about 525 nm. The absorption toward the red is punctuated by several narrow peaks between 541 and 595 nm (and disrupted by two artifacts caused by the pump laser at 527 and 532.5 nm), as listed in detail in Table 1. Some of this structure is also preserved in the spectrum of the dimer, as evidenced by the weak features at 555, 582, and 597 nm. The depletion of the anionic complex, phen^–^He_(1–40)_, extends to about 500 nm, with a broader dip centered at 439 nm. At higher wavelengths and in the anionic dimer, the signal-to-noise ratio does not allow any further reliable observations.

Figure 3 provides details on the wavelength range from 750 to 1000 nm. Unfortunately, the laser power is comparatively weak between 680 and 750 nm, making any reliable interpretations in this region impossible. Above 750 nm, the absorption pattern of phen^+^ becomes very pronounced, with 13 narrow absorption peaks, as listed in Table 1. The protonated phen, in contrast, shows no absorption features above noise in this region. Hydrogenation of PAHs commonly disrupts their aromatic conjugation, opening the possibility of aliphatic behavior and leading to strong red-shifts in their absorption compared to their neutral or radical, cationic counterparts.^26,81–84^

Phen^+^ exhibits another strong absorption at 892 nm (attributed to the D2 ← D0 electronic transition,^85^ while its dimer, phen_2_^+^, exhibits a broad absorption band around 840 nm, and a further, weaker absorption at 878 nm. The anionic phen^–^He_(1–40)_ shows broad absorption detected via depletion with local maxima around 822, 837, 860, 917, and 982 nm. Its dimer, phen_2_^–^He_(1–40)_, reproduces this general trend.

The spectroscopy of the cationic phen in this work shows good agreement with previous studies using the matrix isolation technique, as shown in Table 1. The data presented by Andrews et al.^56^ were obtained from a 20 K argon matrix, whereas Salama et al.^58^ performed their spectroscopy in a 4 K neon matrix. The interaction with the host-matrix naturally introduces a red shift of the absorption lines, which is smallest in the case of liquid helium and becomes minimal when only a few He tags are added. For PAH ions, the attachment of individual He atoms generally produces small, approximately systematic shifts with their magnitude and direction depending on the electronic state and the number of binding sites for the attached He atoms.^63,86^ In the present case, comparison of the phen^+^ spectrum with available gas-phase photofragment spectra indicates shifts of only a few tenths of a nanometer for individual bands. While our data are uniformly shifted to lower wavelengths compared to matrix isolation, the magnitude of this blue shift is not constant across experiments. This can be attributed to state-specific changes in the polarizability of the solid matrix or the He-tags. In this context, HNDs provide a further advantage of our technique, as they perturb the embedded molecules substantially less than any solid matrix can, bringing the results closer to gas-phase conditions. This might also explain the partially better resolved peaks presented in this work, similar to what was previously reported by Bréchignac and coworkers in their gas-phase photofragment spectroscopy in the 760–900 nm region.^85^ Within the range studied therein, our data show excellent agreement with the previously published gas-phase study. In this comparison, individual peaks from our He-tagging spectra show a weak red shift (in the order of a few tenths of nm) of the electronic bands, which might be attributed to the dispersion effects of the He-microsolvation and the resulting stabilization of the more polarizable excited states.^17^

Garkusha and co-workers^26^ provide electronic spectra of protonated anth and phen, detecting three possible isomers of H^+^anth and five isomers of H^+^phen in 6 K neon matrices based on comparison with TD-DFT calculations. Matching peak positions are denoted in Table 1. The experimental spectra of H^+^anth show good agreement between the data from Garkusha et al. and this work, and the transitions can be assigned reasonably well by applying a nearly constant shift to the computations.^26^ H^+^phen, however, does not permit a similarly straightforward assignment and shows several discrepancies in comparison with our spectra. This difference can be explained consistently by the calculations of Saed and Omidyan.^87^ The S_0_(0) ← S_1_(0) transition was calculated to occur at approximately 593 nm, in good agreement with our measured spectrum. Their calculations further show that the ions exhibit significant out-of-plane distortion in the excited S_1_ and S_2_ states, which facilitates rapid relaxation to the ground state via a conical intersection. The structural changes accompanying electronic excitation of H^+^phen result in weak and extended Franck–Condon progressions, a phenomenon that is also known to be present in neutral phenanthrene derivatives.^88–90^ The broad, unstructured absorption observed in the wavelength range up to 600 nm observed here is consistent with these predicted structural changes and nonradiative decay pathways, although the present action spectra do not directly probe the relaxation dynamics.

While this theoretical prediction is consistent with our experimental findings, it differs considerably from results obtained by matrix isolation spectroscopy, where well-resolved, relatively narrow absorption bands have been reported.^26^ In rare-gas solids, extreme geometrical deformations are hindered, which might hinder the out-of-plane deformation of an excited state of the originally planar PAH. This constraint may hinder the structural twisting suggested by Saed and Omidyan^87^ in the excited state, thereby suppressing the relaxation via a conical intersection responsible for the spectral broadening.

In contrast to anth^–^, for which electronically excited resonances have been characterized in detail, the literature on phen^–^ covers mostly its electron affinity and some photoelectron spectroscopy. Resolved electronic absorption spectroscopy is arguably not feasible due to the marginal stability of the bare phen^–^.^41^ As even the He-tagged species sit close to the detachment threshold, optical excitation will rapidly couple to electron detachment. Consequently, the present broad depletions of the action spectrum are best interpreted as weakly bound or resonance-like excited states. This is in line with studies from Lee et al.^36,37^ reporting that the extra electron is fully delocalized over the molecular framework, which leads to a change in its geometry and produces large Franck–Condon envelopes rather than sharp vibronic progressions. In other words, the broad, unresolved action spectrum supports the idea that the singly occupied molecular orbital (SOMO) of phen^–^ is not just a weak perturbation of the neutral π-system, but drives noticeable redistribution of the electron density and altered bond-length patterns in comparison to the neutral phen.

### Dimer Formation

The dimers of anth were thoroughly discussed previously.^77^ Therefore, we will primarily focus on the formation of phen_2_^+^ and H^+^phen_2_ and their spectral properties in the visible, as presented in Figure 4. Our calculations predict a binding energy of about 11064 K for the most stable dimer of phen_2_^+^. This energy is about four to five times smaller than an average C–H chemical bond. Therefore, such clusters are expected to be stable only at relatively low temperatures, in the absence of energetic collisions. The phen molecules are arranged in a π-stacked shifted geometry with some C atoms of one phen positioned above the middle of the aromatic rings of the other phen, thus resembling the orientation of graphene layers in graphite. The molecules are rotated in their plane relative to one another, producing different isomers that differ mainly by the angle of this rotation. The difference in the energies of the isomers is minimal, amounting to only a few hundred kelvins. Therefore, the coexistence of several isomers can be expected.

**Figure 4:**
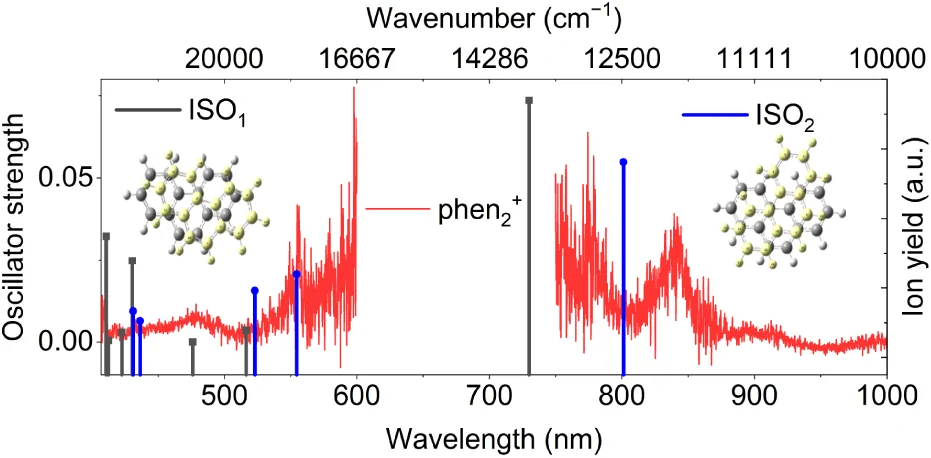
Experimental spectra of the cationic phen dimers (red, transparent data) in comparison to the calculated electronic transitions of two isomers of the phen_2_^+^ complex (gray sticks for the lowest energy isomer ISO_1_, blue sticks for ISO_2_ with Δ*E* = 637 K). The inset shows the structures of both isomers.

The hydrogenation of these dimers proceeds similarly to that of the hydrogenation of monomeric molecules.^26^ The H atom becomes chemically bonded to different C atoms of phen, considerably increasing the number of isomers. Again, the energy difference between different H atom binding sites is small, so that the coexistence of all isomers in the experiment can be expected.^26^

At the same time, different isomers are characterized by different electronic spectra, with the position of the absorption bands differing by a few tens of nanometers. This increases the number of absorption bands and reduces the intensity of the bands associated with the specific isomers. Formation of dimers can also result in broadening of the absorption bands due to several reasons. First, the dimers experience a comparatively larger geometry change in the electronically excited states, leading to a longer Franck–Condon envelope. Furthermore, the dimers are expected to have strong coupling between electron and nuclear motions, resulting in a shortened lifetime of the excited states and leading to considerable natural broadening. A similar congestion of the electronic transitions has been observed in phenanthrene derivates^91,92^ and studies on stacked benzene dimers also suggest considerable changes in the potential energy surface in comparison to the monomer due to their increased charge-resonance character.^93^

These effects can explain the overall decrease in the amplitude of the absorption upon formation of the clusters. However, a part of this reduction is due to the dissociation of the weakly bonded complexes upon energetic photon absorption.^77^

Calculations for three low-lying phen_2_^–^ anion isomers, compare Figure 5, predict a manifold of weakly to moderately allowed electronic transitions extending from the visible into the near-IR. The lowest-energy structures differ by only about 0.02–0.06 eV, such that several isomers may contribute under the experimental conditions. As shown in Figure 5, several calculated transitions lie in the 0.8–1.4 eV range, consistent with the broad depletion observed experimentally between roughly 800 and 1000 nm. Previous PES studies found that the excess electron is delocalized over the dimeric core, changing its electronic structure and possibly stabilizing different detachment channels.^36,37^ Here, the absence of a single dominant bright transition and the overlap of several weak excitations support our interpretation of the anion signal as a broad electronic continuum shaped by isomer coexistence and rapid autodetachment.

**Figure 5:**
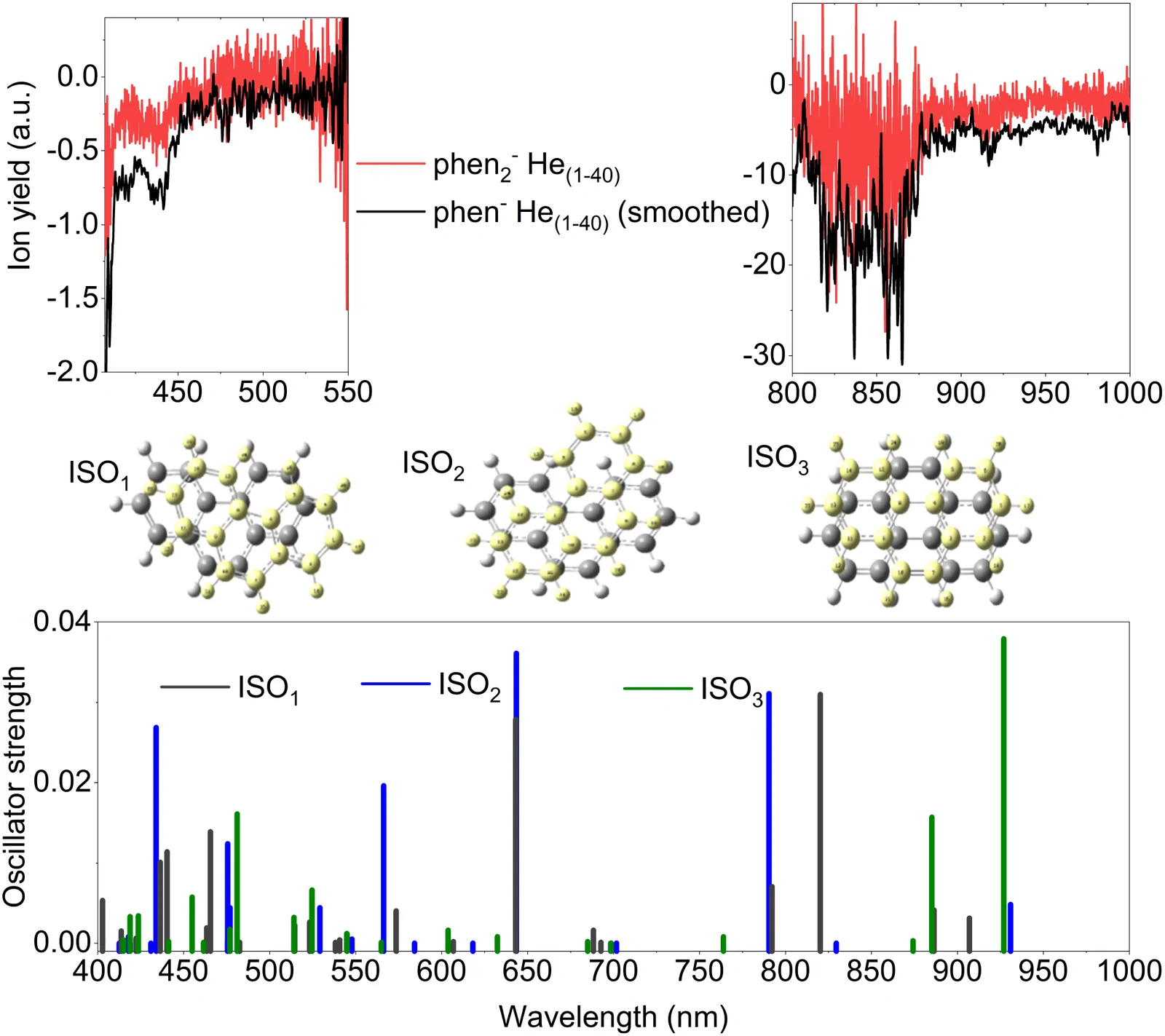
Upper panel: experimental spectra of the anionic phen monomers (black data, smoothed to guide the eye) and dimers (red data). Lower panel: calculated electronic transitions of three isomers of the phen_2_^–^ complex (gray sticks for the lowest energy isomer ISO_1_, blue sticks for ISO_2_ with Δ*E* = 259 K), green sticks for ISO_3_ with Δ*E* = 697 K.

### Geometry-Dependent Trends

This section highlights the most prominent difference between the linear anth and the angular phen complexes. As discussed previously by Meyer et al.,^63^ anth^+^ exhibits strong absorption bands in the 500–750 nm region, which are typical of the vibronic structure of the D_2_ ← D_0_ transition (origin around 720 nm) in linear, cationic PAHs.^25,54,63,85,94^ These low-lying transitions are commonly further red-shifted^25,54^ in the bent phen^+^ framework, as seen in the pronounced absorption peaks in Figure 3. In energetically higher transitions, however, the lower symmetry and more localized electronic structure on the bent phen^+^ increases the oscillator strength in higher excited (doublet) states and opens more vibronic coupling channels.^8,20,23^ An example is the distinct feature around 470 nm attributed to the *D*_4_ ← *D*_0_ transition^56,58^ of phen^+^, that is not found in anth^+^, see Figure 6.

**Figure 6:**
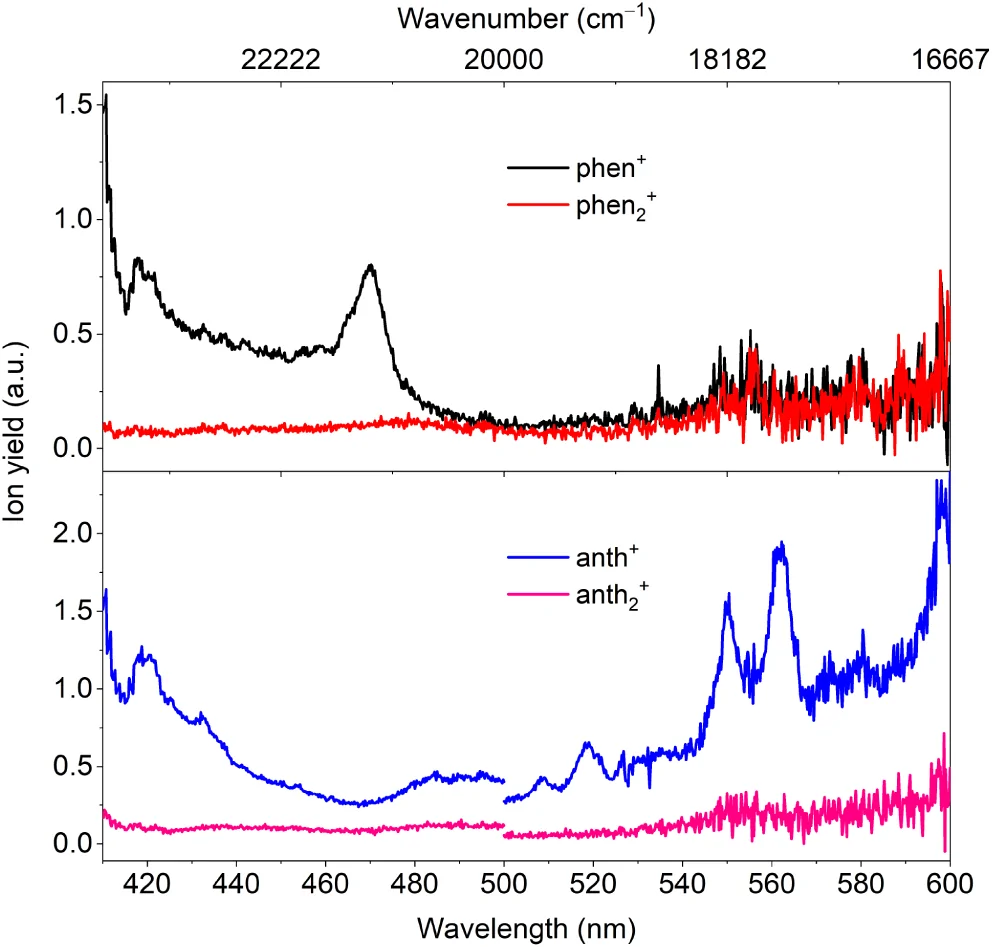
Comparison of the spectra of cationic phen (upper panel, phen^+^ in black and phen_2_^+^ in red) and anth (lower panel, anth^+^ in blue and anth_2_^+^ in magenta) in the range of 410–600 nm.

Dimerization of the complexes enhances these topological tendencies. The anth dimer might allow for a weak exciton-type coupling between the stacked monomers^55,95,96^ and preserve the vibronic progression, while even broader absorption bands with reduced vibronic resolution of phen_2_^+^ likely indicate enhanced nonradiative decay due to stronger, intramolecular vibronic coupling.^23,97^

A complementary trend is observed when comparing the protonated complexes in Figure 7. Hydrogenation disrupts the aromatic π-conjugation and lowers the molecular symmetry of the complexes. The H^+^phen spectrum considerably gains intensity and, to some regard, also structure, especially in the 500–600 nm region, while H^+^anth has an overall weakened oscillator strength and largely quenched vibronic structure, with some remnant modulation below 500 nm. The angular structure of phen likely allows the added proton to be localized more effectively within a specific region of the π-framework, preserving or even enhancing localized transitions^25^ and giving rise to additional relaxation channels via conical intersections.^87^ Beyond 650 nm, the signal decays rapidly in both systems, likely reflecting changes in the accessible electronic transitions and/or competing relaxation pathways. However, the present action spectra alone do not allow for a clear distinction of these effects.

**Figure 7:**
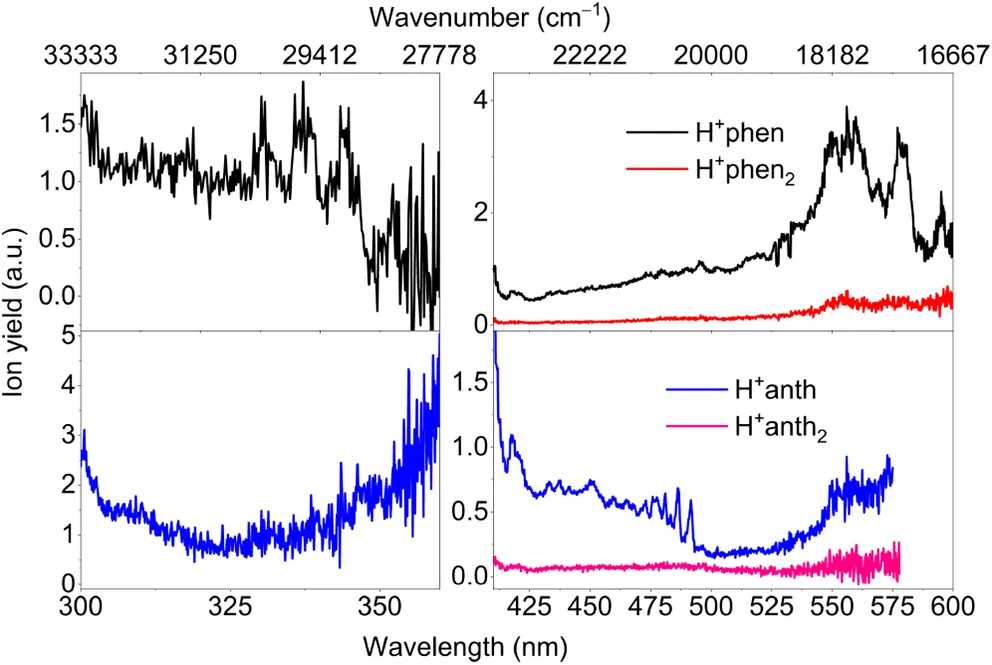
Comparison of protonated phen (top panel) and anth (lower panel) and their dimers. The hydrogenation disrupts the π-bonds in the complexes, thereby quenching the vibronic fine structure. This effect is more pronounced for the linear anth, which loses conjugation, whereas the additional H is more localized within the bent phen, which can, in turn, retain some intense absorption bands.

## Conclusion

The phenanthrene spectra of this work reveal a clear charge-dependent progression, from anionic continua dominated by autodetachment to intense cationic vibronic bands and, for H^+^phen, broader spectral features consistent with more localized electronic transitions and efficient nonradiative relaxation, underscoring how ionization modulates the localization and optical activity of the bent π-system. The contrast between anth and phen across all charge states highlights how molecular geometry dictates the electronic response of PAH oligomers. Linear anth ions preserve delocalized, vibronically structured transitions that broaden and weaken upon dimerization or hydrogenation. The angular phen ions display intrinsically broader bands, which can be regarded as a signature of enhanced vibronic coupling, charge localization, and partial internal conversion in their bent π-framework. In particular, the broad absorption of H^+^phen agrees with the out-of-plane twisted excited-state geometry predicted by Saed and Omidyan^87^ and its associated rapid nonradiative relaxation. The contrast with the narrower matrix-isolation spectra further suggests that rare-gas confinement suppresses the large-amplitude out-of-plane distortion and associated conical-intersection-mediated relaxation of excited H^+^phen. This interplay of electronic delocalization, excitonic coupling, and nonradiative relaxation across both isomers is essential for interpreting astrochemical spectra and modeling charge-transfer processes in aromatic materials and can hence provide experimental benchmark data for the photophysics of ionized PAH isomers. Extending He-tagging action spectroscopy to larger and substituted PAHs will allow these topology-dependent trends to be tested across a broader range of molecular structures and may help clarify how molecular size, edge structure, and functionalization influence charge localization and the resulting electronic spectra.

## Supplementary Material



## Data Availability

The data underlying this article are available in the Zenodo repository under the DOI 10.5281/zenodo.21128824.

## Notes

Calculated structures and electronic excited states of protonated phenanthrene and cationic and anionic phenanthrene dimers; Cartesian coordinates of the optimized dimer structures (PDF)
