# Supplementary material for: Topology-Controlled Photophysics of Ionized C14H10 Isomers
Source: J Phys Chem A. 2026 Sep 21;130(39):7812–21. doi: 10.1021/acs.jpca.6c04463 (PMC13637018; doi:10.1021/acs.jpca.6c04463)
Supplement: Supplementary file 1 [file jp6c04463_si_001.pdf]

Topology-Controlled Photophysics of Ionized $C_{14}H_{10}$ Isomers

A. M. Reider[1,*], M. Kappe[1], P. Martini[2], M. Schmidt[1], S. A. Krasnokutski[3,**], and P. Scheier[1]

[1]University of Innsbruck, Technikerstrasse 25, 6020 Innsbruck, Austria
[2]University of Stockholm, AlbaNova, Roslagstullsbacken 21, 11421 Stockholm, Sweden
[3]University of Jena, Helmholtzweg 3, 07743 Jena, Germany

*Email: anna-maria.reider@uibk.ac.at
**Email: sergiy.krasnokutskiy@uni-jena.de

Supporting Information

Protonated Phenanthrene

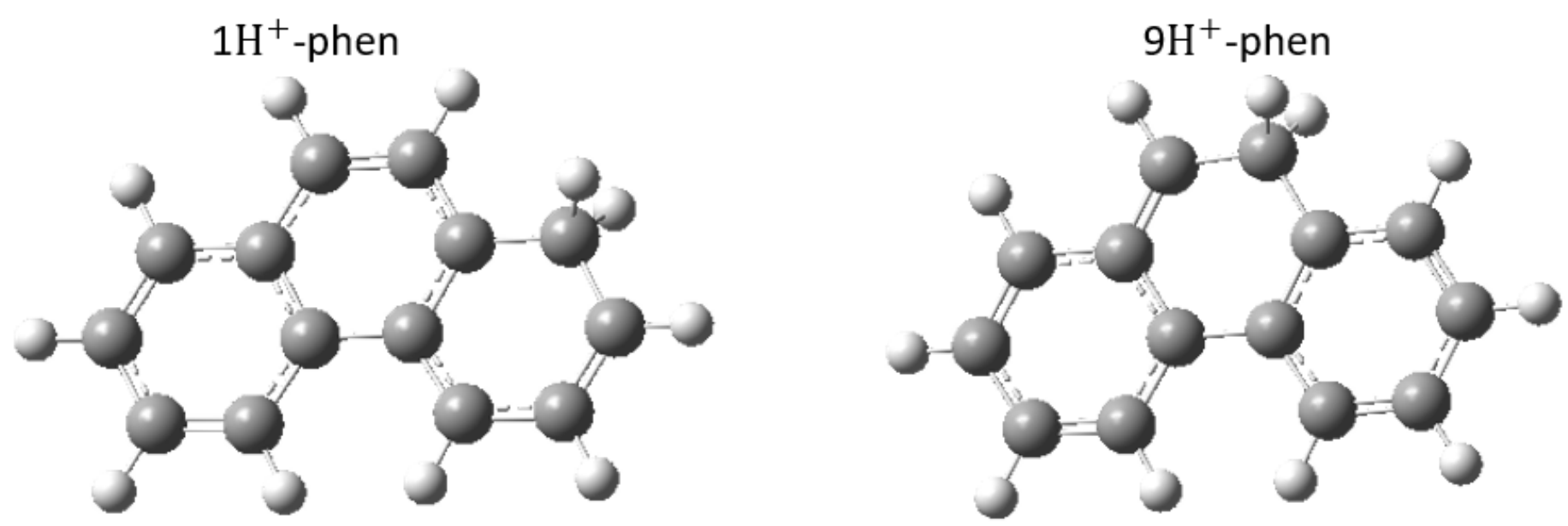

Figure S1: The two lowest-lying isomers found in the computations of H^+phen with the position of the additional H^+ denoting its nomenclature.

Table S1: Calculated electronic excited states of the first isomer, 1H-phen$^+$ with $\Delta E = 245$ K, of redprotonated phen. Excitation energies, wavelengths, oscillator strengths, and spin expectation values are listed.

State	Symmetry	E (eV)	λ (nm)	f
1	Singlet-A	2.7463	451.45	0.1626
2	Singlet-A	3.2014	387.28	0.0286
3	Singlet-A	4.4291	279.93	0.0198
4	Singlet-A	4.7404	261.55	0.0957
5	Singlet-A	5.0555	245.24	0.0001
6	Singlet-A	5.2687	235.32	0.0409
7	Singlet-A	5.3204	233.04	0.0001
8	Singlet-A	5.3784	230.52	0.5041
9	Singlet-A	6.0914	203.54	0.0012
10	Singlet-A	6.1002	203.25	0.1172

Dimers of Phenanthrene

Note: The $\langle S^2 \rangle$ values indicate increasing spin contamination for several higher-lying states.

Table S2: Calculated electronic excited states of the second isomer, 9H-phen$^+$ with $\Delta E = 245$ K, of redprotonated phen. Excitation energies, wavelengths, oscillator strengths, and spin expectation values are listed.

State	Symmetry	E (eV)	λ (nm)	f	$\langle S^2 \rangle$
1	Singlet-A	2.7810	445.83	0.2415	0.000
2	Singlet-A	3.1342	395.59	0.0238	0.000
3	Singlet-A	4.3106	287.63	0.1016	0.000
4	Singlet-A	4.8756	254.30	0.0194	0.000
5	Singlet-A	5.0178	247.09	0.0098	0.000
6	Singlet-A	5.2041	238.24	0.0000	0.000
7	Singlet-A	5.2500	236.16	0.0015	0.000
8	Singlet-A	5.7009	217.48	0.0263	0.000
9	Singlet-A	5.9399	208.73	0.4941	0.000
10	Singlet-A	6.1150	202.76	0.3386	0.000

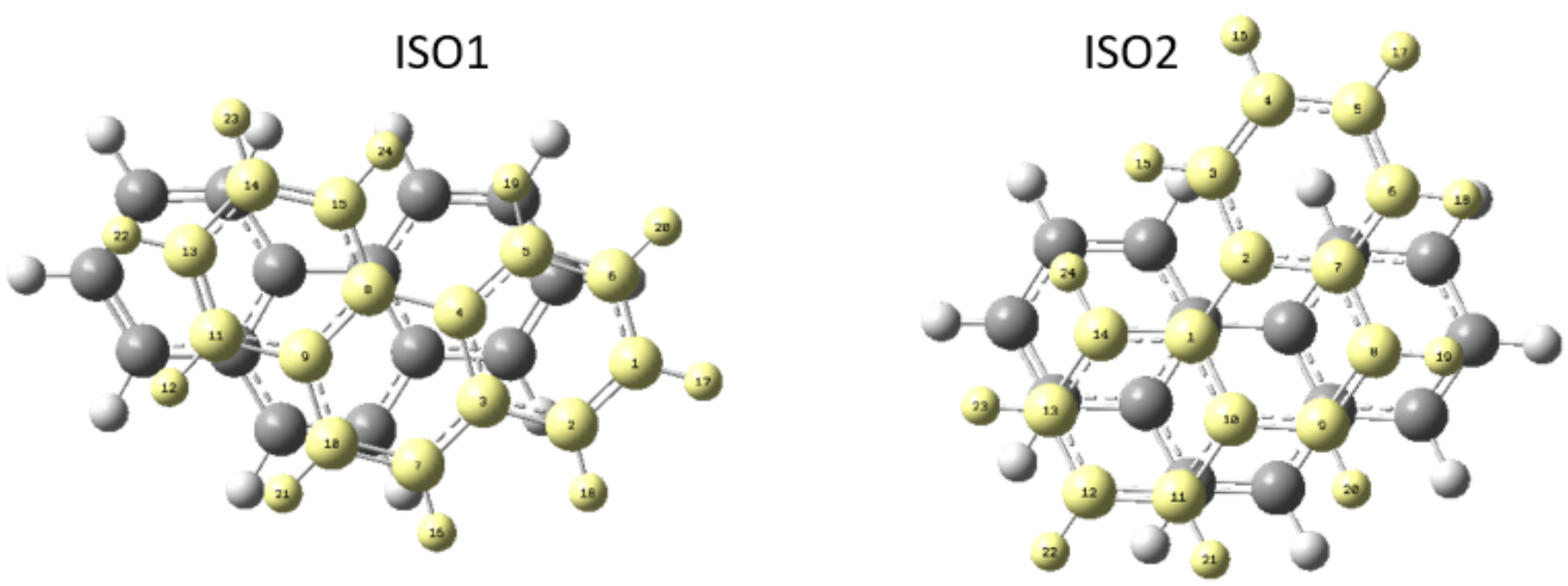

Figure S2: The two lowest-lying isomers found in the computations of $phen_2{}^+$.

Table S3: Calculated electronic excited states of the first isomer, ISO_1 with $\Delta E = 637$ K, of cationic $phen_2{}^+$. Excitation energies, wavelengths, oscillator strengths, and spin expectation values are listed.

State	State label	E (eV)	λ (nm)	f	$\langle S^2 \rangle$
1	2.033-A	0.9244	1341.28	0.1148	0.784
2	2.040-A	1.0471	1184.02	0.0095	0.791
3	2.044-A	1.0594	1170.32	0.0000	0.794
4	2.038-A	1.6983	730.04	0.0736	0.789
5	2.097-A	2.4006	516.47	0.0036	0.850
6	2.119-A	2.6044	476.05	0.0000	0.873
7	3.193-A	2.8801	430.49	0.0248	2.299
8	2.904-A	2.9329	422.74	0.0030	1.859
9	2.194-A	3.0056	412.50	0.0005	0.954
10	2.211-A	3.0177	410.85	0.0323	0.972
11	3.322-A	3.7449	331.08	0.0016	2.509
12	3.262-A	3.7607	329.68	0.0003	2.410
13	3.079-A	3.7687	328.99	0.0003	2.121
14	3.169-A	3.7908	327.07	0.0157	2.260
15	3.071-A	3.8280	323.89	0.0021	2.108

Table S4: Calculated electronic excited states of the second isomer, ISO_2 with $\Delta E = 637$ K, of $phen_2{}^+$. Excitation energies, wavelengths, oscillator strengths, and spin expectation values are listed.

State	State label	E (eV)	λ (nm)	f	$\langle S^2 \rangle$
1	2.022-A	0.6188	2003.78	0.0671	0.773
2	2.031-A	0.9267	1337.92	0.0005	0.782
3	2.035-A	1.0208	1214.56	0.0122	0.785
4	2.026-A	1.5474	801.22	0.0548	0.776
5	2.058-A	2.2355	554.60	0.0206	0.809
6	2.076-A	2.3704	523.05	0.0156	0.828
7	3.200-A	2.8406	436.48	0.0064	2.311
8	3.058-A	2.8754	431.20	0.0094	2.088
9	2.183-A	3.0791	402.67	0.0000	0.941
10	2.226-A	3.3594	369.07	0.0138	0.988
11	2.425-A	3.4783	356.45	0.0054	1.220
12	3.154-A	3.7173	333.54	0.0027	2.238
13	3.176-A	3.7702	328.85	0.0038	2.272
14	3.343-A	3.7742	328.50	0.0010	2.544
15	3.176-A	3.8049	325.85	0.0005	2.271

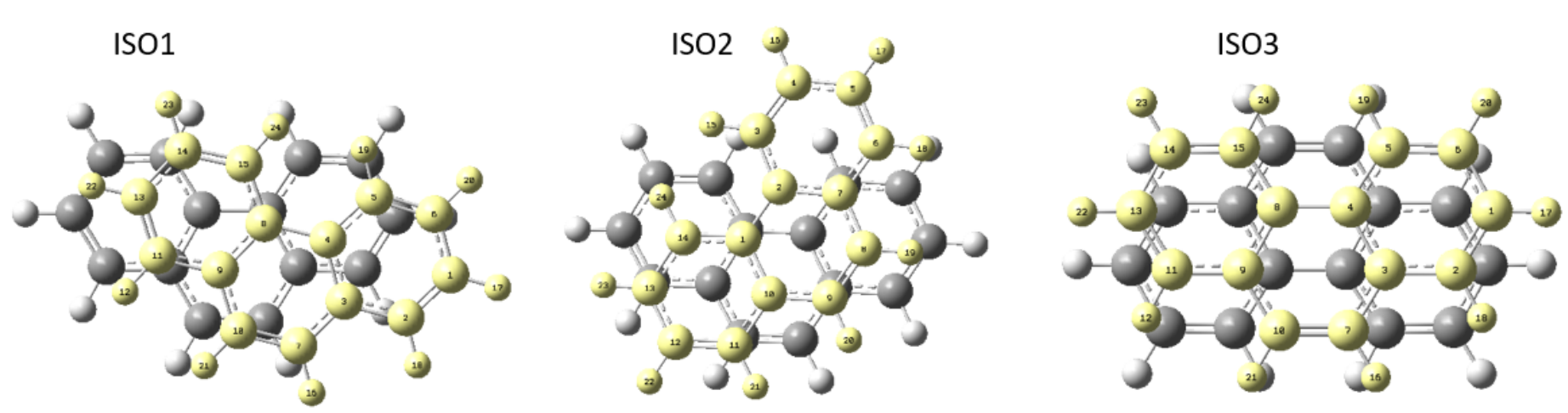

Figure S3: The three lowest-lying isomers found in the computations of $phen_2{}^-$.

Table S5: Calculated electronic excited states of the first isomer, ISO_1 with ΔE = 259 K, of $phen_2{}^-$. Excitation energies, wavelengths, oscillator strengths, and spin expectation values are listed.

State	State label	E (eV)	λ (nm)	f	$\langle S^2 \rangle$
1	2.022-A	0.4390	2824.15	0.0156	0.772
2	2.020-A	0.6873	1804.02	0.0765	0.771
3	2.029-A	0.9207	1346.63	0.0007	0.779
4	2.020-A	1.2165	1019.15	0.0004	0.770
5	2.021-A	1.3375	926.96	0.0379	0.771
6	2.021-A	1.4007	885.18	0.0157	0.771
7	2.023-A	1.4183	874.15	0.0003	0.773
8	2.024-A	1.6231	763.89	0.0008	0.774
9	2.021-A	1.7747	698.63	0.0000	0.771
10	2.022-A	1.8103	684.87	0.0002	0.772
11	2.025-A	1.9605	632.43	0.0008	0.775
12	2.024-A	2.0534	603.80	0.0016	0.774
13	2.027-A	2.1946	564.94	0.0001	0.778
14	2.037-A	2.2747	545.05	0.0011	0.787
15	2.025-A	2.2757	544.82	0.0012	0.776
16	2.040-A	2.3633	524.63	0.0066	0.790
17	2.060-A	2.4115	514.14	0.0032	0.811
18	2.114-A	2.5772	481.09	0.0161	0.868
19	2.034-A	2.5998	476.91	0.0017	0.784
20	2.291-A	2.6861	461.58	0.0001	1.062
21	3.050-A	2.7257	454.87	0.0057	2.076
22	2.912-A	2.8100	441.23	0.0002	1.870
23	2.027-A	2.9267	423.63	0.0034	0.777
24	2.034-A	2.9604	418.82	0.0033	0.785
25	2.026-A	2.9880	414.94	0.0004	0.776

Table S6: Calculated electronic excited states of the second isomer, ISO_2 with ΔE = 259 K, of $phen_2{}^-$. Excitation energies, wavelengths, oscillator strengths, and spin expectation values are listed.

State	State label	E (eV)	λ (nm)	f	$\langle S^2 \rangle$
1	2.020-A	0.6238	1987.47	0.0628	0.771
2	2.023-A	0.6686	1854.38	0.0010	0.773
3	2.025-A	0.7332	1691.11	0.0227	0.775
4	2.021-A	1.2373	1002.05	0.0002	0.771
5	2.022-A	1.3670	906.99	0.0031	0.772
6	2.022-A	1.3988	886.37	0.0041	0.772
7	2.020-A	1.5115	820.26	0.0310	0.770
8	2.025-A	1.5651	792.18	0.0070	0.775
9	2.022-A	1.7900	692.64	0.0001	0.772
10	2.022-A	1.8014	688.28	0.0016	0.772
11	2.023-A	1.9280	643.08	0.0279	0.773
12	2.024-A	2.0432	606.82	0.0002	0.774
13	2.023-A	2.1615	573.61	0.0040	0.773
14	2.024-A	2.2930	540.70	0.0004	0.774
15	2.033-A	2.3034	538.26	0.0001	0.784
16	2.042-A	2.3694	523.27	0.0026	0.793
17	2.054-A	2.4093	514.61	0.0022	0.804
18	2.040-A	2.5693	482.56	0.0001	0.791
19	2.034-A	2.6634	465.51	0.0139	0.784
20	2.128-A	2.6755	463.40	0.0019	0.883
21	3.119-A	2.8155	440.36	0.0114	2.183
22	2.988-A	2.8413	436.36	0.0101	1.982
23	2.031-A	2.9365	422.22	0.0006	0.781
24	2.032-A	2.9974	413.64	0.0015	0.782
25	2.043-A	3.0780	402.81	0.0053	0.793

Table S7: Calculated electronic excited states of the third isomer, ISO_3 with $\Delta E = 697$ K, of $phen_2{}^-$. Excitation energies, wavelengths, oscillator strengths, and spin expectation values are listed.

State	State label	E (eV)	λ (nm)	f	$\langle S^2 \rangle$
1	2.024-A	0.5664	2189.00	0.0000	0.774
2	2.023-A	0.6377	1944.31	0.0811	0.773
3	2.024-A	0.8080	1534.50	0.0079	0.774
4	2.026-A	1.2280	1009.64	0.0000	0.776
5	2.024-A	1.3319	930.86	0.0048	0.774
6	2.027-A	1.3990	886.22	0.0029	0.778
7	2.035-A	1.4947	829.50	0.0000	0.786
8	2.023-A	1.5687	790.39	0.0311	0.773
9	2.025-A	1.7670	701.67	0.0000	0.775
10	2.027-A	1.7754	698.36	0.0000	0.777
11	2.028-A	1.9268	643.46	0.0361	0.778
12	2.027-A	2.0055	618.22	0.0000	0.777
13	2.027-A	2.1220	584.27	0.0000	0.777
14	2.059-A	2.1892	566.34	0.0196	0.809
15	2.027-A	2.2630	547.87	0.0005	0.777
16	2.032-A	2.3427	529.23	0.0044	0.782
17	2.084-A	2.5701	482.41	0.0000	0.836
18	2.041-A	2.5997	476.93	0.0044	0.791
19	2.084-A	2.6068	475.61	0.0124	0.836
20	2.028-A	2.6076	475.47	0.0003	0.778
21	3.112-A	2.8573	433.93	0.0269	2.172
22	3.125-A	2.8772	430.92	0.0000	2.191
23	2.027-A	2.9599	418.88	0.0000	0.777
24	2.042-A	2.9668	417.90	0.0008	0.792
25	2.077-A	3.0058	412.49	0.0000	0.828

Table S8: Cartesian coordinates for ISO1+

Atom	X (Å)	Y (Å)	Z (Å)
C	4.455371	0.401317	0.133616
C	3.622261	0.596737	1.207876
C	2.278385	0.992182	1.019143
C	1.774333	1.182810	-0.294462
C	2.644177	0.971456	-1.366115
C	3.959693	0.592375	-1.158277
C	1.432639	1.203765	2.133018
C	0.387262	1.598410	-0.468102
C	-0.417443	1.810883	0.681734
C	0.126915	1.594464	1.968914
C	-1.759420	2.233671	0.537154
H	-2.351833	2.401622	1.428579
C	-2.298894	2.438806	-0.704541
C	-1.509923	2.217371	-1.839534
C	-0.194202	1.810582	-1.722129
H	1.834038	1.063441	3.130126
H	5.486727	0.109362	0.284112
H	3.989474	0.462484	2.218935
H	2.305105	1.113681	-2.382439
H	4.611695	0.447313	-2.011005
H	-0.496952	1.770869	2.837127
H	-3.325198	2.764648	-0.811239
H	-1.929716	2.380659	-2.824685
H	0.382543	1.663460	-2.624461
C	2.298884	-2.438723	-0.705061
C	1.759272	-2.234218	0.536670
C	0.417330	-1.811409	0.681359
C	-0.387352	-1.598558	-0.468434
C	0.194274	-1.810030	-1.722512
C	1.510035	-2.216676	-1.840008
C	-0.126971	-1.595022	1.968583
C	-1.774431	-1.183072	-0.294684
C	-2.278446	-0.992518	1.018910
C	-1.432641	-1.204179	2.132740
C	-3.622219	-0.596708	1.207691
H	-3.989300	-0.462229	2.218769
C	-4.455299	-0.401061	0.133466
C	-3.959594	-0.591931	-1.158459
C	-2.644145	-0.971167	-1.366346
H	0.496887	-1.771570	2.836774
H	3.325182	-2.764547	-0.811850
H	2.351642	-2.402528	1.428058
H	-0.382345	-1.662486	-2.624862
H	1.929943	-2.379370	-2.825211
H	-1.833968	-1.063834	3.129876
H	-5.486580	-0.108857	0.283986
H	-4.611537	-0.446468	-2.011166
H	-2.304930	-1.112988	-2.382675

Table S9: Cartesian coordinates for ISO2+

Atom	X (Å)	Y (Å)	Z (Å)
C	-0.160807	-0.166961	1.707616
C	1.080094	0.561427	1.486582
C	1.174384	1.956581	1.572057
C	2.370595	2.606167	1.346433
C	3.527053	1.887780	1.019971
C	3.466601	0.520478	0.927133
C	2.253984	-0.166591	1.159532
C	2.201944	-1.578796	1.060609
C	1.035888	-2.263169	1.271107
C	-0.162287	-1.580924	1.588977
C	-1.364987	-2.299844	1.778293
C	-2.537320	-1.650073	2.101199
C	-2.534349	-0.264002	2.220143
C	-1.367227	0.462693	2.016216
H	0.305329	2.548049	1.824340
H	2.415325	3.685234	1.430025
H	4.460778	2.408928	0.851210
H	4.352916	-0.054256	0.685053
H	3.110795	-2.118185	0.822313
H	1.019547	-3.344168	1.194455
H	-1.344893	-3.380000	1.689624
H	-3.448051	-2.212179	2.263760
H	-3.446481	0.260502	2.475000
H	-1.415688	1.539171	2.100425
C	-1.083820	0.810402	-1.373388
C	0.160675	0.135254	-1.711872
C	1.362611	0.814042	-1.915522
C	2.533938	0.136729	-2.234286
C	2.545130	-1.250030	-2.342290
C	1.376757	-1.950633	-2.129629
C	0.170469	-1.279042	-1.825470
C	-1.023795	-2.010632	-1.624191
C	-2.194181	-1.376460	-1.306476
C	-2.254077	0.032151	-1.171951
C	-3.470261	0.665050	-0.829279
C	-3.537381	2.028237	-0.692597
C	-2.384211	2.796458	-0.893574
C	-1.185049	2.199623	-1.224723
H	1.404394	1.890319	-1.825423
H	3.442635	0.701048	-2.401159
H	3.458987	-1.772827	-2.594004
H	1.362772	-3.030840	-2.218580
H	-1.001339	-3.089534	-1.724969
H	-3.099892	-1.952894	-1.161127
H	-4.353501	0.053488	-0.686700
H	-4.473560	2.509546	-0.440017
H	-2.433419	3.873995	-0.794215
H	-0.318883	2.829109	-1.373726

Table S10: Cartesian coordinates for $ISO1^-$

Atom	X (Å)	Y (Å)	Z (Å)
C	4.229869	0.709140	0.336529
C	3.333739	0.870488	1.361162
C	1.980622	1.215505	1.118159
C	1.553875	1.384036	-0.232944
C	2.495017	1.200490	-1.253342
C	3.811510	0.875084	-0.992990
C	1.060164	1.386061	2.177344
C	0.160360	1.744032	-0.493807
C	-0.723738	1.908229	0.608136
C	-0.248528	1.722823	1.935972
C	-2.074197	2.236243	0.352590
H	-2.750169	2.325938	1.195654
C	-2.542739	2.411730	-0.928926
C	-1.674763	2.261790	-2.011338
C	-0.350199	1.927539	-1.785772
H	1.408964	1.235291	3.193578
H	5.259077	0.440881	0.552649
H	3.650830	0.732152	2.390432
H	2.187607	1.300390	-2.286215
H	4.508532	0.731367	-1.810277
H	-0.949208	1.838784	2.754854
H	-3.589084	2.641139	-1.097342
H	-2.037882	2.383944	-3.025638
H	0.299845	1.795602	-2.641174
C	2.543121	-2.406637	-0.940382
C	2.073874	-2.239750	0.341983
C	0.723291	-1.912874	0.599105
C	-0.160003	-1.740878	-0.502458
C	0.351353	-1.915657	-1.795195
C	1.675979	-2.248878	-2.022372
C	0.247284	-1.736437	1.927649
C	-1.553735	-1.382209	-0.240149
C	-1.981314	-1.222650	1.111920
C	-1.061736	-1.400718	2.170423
C	-3.334586	-0.878564	1.356069
H	-3.652434	-0.746855	2.385989
C	-4.229888	-0.710049	0.331823
C	-3.810732	-0.867317	-0.998441
C	-2.493985	-1.191449	-1.259857
H	0.947207	-1.858522	2.746310
H	3.589466	-2.635491	-1.109666
H	2.749287	-2.335687	1.184829
H	-0.298065	-1.777528	-2.650108
H	2.039672	-2.364171	-3.037262
H	-1.411186	-1.256960	3.187464
H	-5.259136	-0.442739	0.549003
H	-4.507052	-0.717875	-1.815295
H	-2.185758	-1.284407	-2.293147

Table S11: Cartesian coordinates for $ISO2^{-}$

Atom	X (Å)	Y (Å)	Z (Å)
C	-0.673705	-1.705621	0.038306
C	0.654801	-1.622031	0.644086
C	0.851614	-1.538811	2.026436
C	2.117768	-1.460038	2.583168
C	3.243403	-1.471190	1.748464
C	3.086692	-1.527652	0.386840
C	1.800241	-1.587174	-0.206517
C	1.637307	-1.588741	-1.612241
C	0.387469	-1.594417	-2.180080
C	-0.782540	-1.666515	-1.379470
C	-2.072106	-1.677946	-1.958959
C	-3.207238	-1.769981	-1.186714
C	-3.098919	-1.840024	0.203303
C	-1.847542	-1.807772	0.794544
H	-0.005399	-1.513379	2.686547
H	2.234066	-1.389603	3.658810
H	4.238147	-1.409011	2.177932
H	3.953029	-1.501960	-0.265479
H	2.523782	-1.541007	-2.234611
H	0.272402	-1.560771	-3.258035
H	-2.150706	-1.613148	-3.039438
H	-4.185205	-1.772725	-1.657203
H	-3.987566	-1.896962	0.820622
H	-1.789269	-1.835703	1.874266
C	-0.654861	1.623633	0.640471
C	0.673733	1.705823	0.034542
C	1.847496	1.809622	0.790554
C	3.099028	1.840390	0.199359
C	3.207401	1.767042	-1.190440
C	2.072305	1.673287	-1.962597
C	0.782617	1.663322	-1.383252
C	-0.387273	1.589357	-2.183670
C	-1.637290	1.584890	-1.615787
C	-1.800292	1.586587	-0.210222
C	-3.086818	1.528416	0.383347
C	-3.243546	1.475133	1.745076
C	-2.117968	1.466182	2.579917
C	-0.851774	1.543806	2.022966
H	1.789158	1.840141	1.870207
H	3.987609	1.898692	0.816650
H	4.185393	1.768629	-1.660901
H	2.151035	1.605999	-3.042919
H	-0.272252	1.553305	-3.261556
H	-2.523693	1.535722	-2.238161
H	-3.953147	1.501404	-0.268935
H	-4.238308	1.414037	2.174679
H	-2.234286	1.398347	3.655722
H	0.005198	1.520528	2.683236

Table S12: Cartesian coordinates for ISO3^{-}

Atom	X (Å)	Y (Å)	Z (Å)
C	3.552573	1.787985	0.160653
C	2.839669	1.334217	1.240410
C	1.421586	1.288935	1.218402
C	0.734665	1.748445	0.058228
C	1.498625	2.186955	-1.032162
C	2.882671	2.210073	-0.994223
C	0.687970	0.787591	2.319788
C	-0.727499	1.750405	0.058247
C	-1.415687	1.292407	1.218263
C	-0.683416	0.789208	2.319746
C	-2.833585	1.340997	1.240017
H	-3.347836	0.986441	2.126541
C	-3.545267	1.797166	0.160408
C	-2.874220	2.218535	-0.993997
C	-1.490253	2.191430	-1.031964
H	1.237463	0.398563	3.170268
H	4.637236	1.797654	0.191485
H	3.352882	0.979071	2.127303
H	1.000863	2.518260	-1.933860
H	3.442903	2.552216	-1.857068
H	-1.233934	0.401423	3.170122
H	-4.629892	1.809247	0.191155
H	-3.433423	2.562735	-1.856686
H	-0.991508	2.521817	-1.933456
C	-3.552438	-1.789245	-0.158952
C	-2.840210	-1.334745	-1.238870
C	-1.422118	-1.289406	-1.217813
C	-0.734486	-1.749309	-0.058144
C	-1.497765	-2.188455	1.032414
C	-2.881850	-2.212095	0.995222
C	-0.689240	-0.787458	-2.319329
C	0.727727	-1.750798	-0.058804
C	1.415202	-1.291551	-1.218850
C	0.682260	-0.788418	-2.319791
C	2.833183	-1.338502	-1.240757
H	3.346926	-0.982050	-2.126810
C	3.545512	-1.794789	-0.161615
C	2.875174	-2.217995	0.992487
C	1.491118	-2.192156	1.030752
H	-1.239209	-0.398382	-3.169478
H	-4.637111	-1.798809	-0.189196
H	-3.354039	-0.979006	-2.125175
H	-0.999378	-2.519970	1.933691
H	-3.441464	-2.554942	1.858182
H	1.232162	-0.399594	-3.170099
H	4.630149	-1.804995	-0.192374
H	3.434864	-2.562454	1.854754
H	0.992934	-2.523793	1.932101
